# Cleft sidedness and congenitally missing teeth in patients with cleft lip and palate patients

**DOI:** 10.1186/s40510-016-0127-z

**Published:** 2016-05-09

**Authors:** Abdolreza Jamilian, Alessandra Lucchese, Alireza Darnahal, Zinat Kamali, Letizia Perillo

**Affiliations:** Department of Orthodontics, Orthognathic Surgery, Tehran Dental Branch, Craniomaxillofacial Research Center, Islamic Azad University, No 2713, Vali Asr St, Tehran, 1966843133 Iran; Department of Orthodontics, Vita-Salute San Raffaele University, Milan, Italy; Department of Orthodontics, Dental Branch, Islamic Azad University, Tehran, Iran; National Nutrition and Food Technology Research Institute, Faculty of Nutrition Sciences and Food Technology, Shahid Beheshti University of Medical Sciences, Tehran, Iran; Orthodontic Unit and Chair of Postgraduate Orthodontic Program, Multidisciplinary Department of Medical-Surgical and Dental Specialties, Second University of Naples, Naples, Italy

**Keywords:** Cleft lip and palate, Cleft sidedness, Missing teeth, Cleft lip and alveolus

## Abstract

**Background:**

The aim of this study was to investigate the prevalence of cleft sidedness, and the number of congenitally missing teeth in regard to cleft type and gender.

**Methods:**

The charts, models, radiographs, and intraoral photographs of 201 cleft patients including 131 males with the mean age of 12.3 ± 4 years and 70 females with the mean age of 12.6 ± 3.9 years were used for the study. *T* test, Chi-square, and binomial tests were used for assessment of the data.

**Results and conclusions:**

One hundred forty-eight of the subjects suffered from cleft lip and palate followed by 41 subjects who suffered from cleft lip and alveolus. Chi-square test did not show any significant difference between the genders. Binomial test showed that left-sided cleft was more predominant in unilateral cleft lip and palate patients (*P* < 0.001). This study also showed that the upper lateral incisors were the most commonly missing teeth in the cleft area.

## Background

Cleft lip and/or palate (CLP) is among the most common congenital human malformations. Failure of fusion of the maxillary and medial nasal prominences or between the palatal processes results in clefts of varying extent, unilaterally or bilaterally. CLP affects between one and seven out of 1000 newborns [1, 2]. The frequency of cleft is higher in Asian people than in other races [3, 4].

Tooth agenesis, also known as hypodontia or congenital absence of teeth, is the most frequently observed developmental anomaly of the human dentition. The prevalence of congenitally missing teeth in the general population has been reported within a range of 0.027 to 10.1 %, which varies greatly according to geographic location and race [5–7]. Clefts of all types are often associated with congenitally missing teeth,[8] and when compared with the general population, subjects with CLP have always been found to have a higher prevalence of dental anomalies, such as variations in tooth number and position, and reduced tooth dimensions, most of which are localized in the area of the cleft defect [9–11]. Rullo et al. examined the prevalence of different types of dental anomalies in children with cleft and found that congenital absence of the cleft-side lateral incisor was observed in 40 % of the samples and a total of 30 % patients showed supernumerary teeth at the incisors region [12].

Baek and Kim [5] investigated the differences in the congenital missing teeth pattern in terms of tooth type and cleft sidedness in Korean CLP patients and found that boys had more congenital missing maxillary lateral incisors on the cleft side than girls, but on the non-cleft side, the congenital absence of the maxillary second premolar was more frequent in girls.

To date, few researches have individually studied the different types of cleft including unilateral or bilateral cleft lip, cleft palate, cleft lip and palate, and cleft alveolus and the incidence of tooth agenesis in each type. Therefore, the aim of this study was to investigate the prevalence of different cleft types, cleft sidedness, and congenitally missing teeth in each cleft type.

## Methods

The study conducted as a follow-up of the study done by Jamilian et al. [13] was carried out in accordance with the ethical standards set forth in the 1964 Declaration of Helsinki. Informed written consent was obtained from each patient and a parent or guardian. Two hundred two consecutive cleft lip and/or palate patients who were referred to orthodontic department of SBUMS from 2009 until 2011 were included in the study. Except for one subject who was excluded from the study, none of the subjects had other known syndromes. Subjects’ distribution according to gender can be seen in Table 1. The final sample of 201 subjects included 131 males with the mean age of 12.3 ± 4 years and 70 females with the mean age of 12.6 ± 3.9 years. The patients’ population was racially and ethnically similar. Lateral cephalograms, OPGs, and photos of patients which were taken for treatment were used for observational purposes of this study. Panoramic and/or periapical and occlusal radiographs of the patients were used to determine the presence or absence of the teeth.

**Table 1:** Gender distribution of samples

Gender	*N* (%)	Age (year) Mean ± SD
Male	131 (65.2)	12.3 ± 4
Female	70 (34.8)	12.6 ± 3.9

Two observers analyzed the records of the patients at the same time. The results of their observations were blinded to each other. No differences were found between the assessments.

The Statistical Package for Social Sciences, Version 20 (SPSS Inc. Chicago, IL, USA) was used to analyze the data. *T* test, Chi-square, and binomial tests were used to analyze the data and *P* value was set at *P* < 0.05.

## Results

Two hundred one consecutive cleft patients including 131 males and 70 females were examined and classified according to their cleft type. Observation of the records showed that the majority of patients suffered from cleft lip and palate (148 subjects), while only three of the subjects suffered just from cleft lip. Further distribution of the subjects according to the type of cleft can be seen in Table 2. Cleft lip patients were not included in the statistical analysis due to the very low number of patients. Although there was a higher tendency for male dominance in the unilateral cleft lip and alveolus and unilateral cleft lip and palate patients, Chi-square test showed that there was no relationship between patients’ sex and the affected side (Table 3) In addition, binomial test showed that the patients suffering from unilateral cleft lip and palate had higher incidence on the left side (*P* < 0.001) (Table 4). As can be seen in Table 5, the incidence of missing teeth in cleft side is higher than the non-cleft side of both unilateral cleft lip and alveolus and unilateral cleft lip and palate subjects.

**Table 2:** Distribution of samples according to cleft type

Gender	Unilateral cleft lip	Bilateral cleft lip	Unilateral cleft lip and alveolus	Bilateral cleft lip and alveolus	Cleft palate	Unilateral cleft lip and palate	Bilateral cleft lip and palate	Total
Male	1	1	18	8	2	64	37	131
Female	–	1	11	4	7	27	20	70
Total	1	2	29	12	9	91	57	201

**Table 3:** Distribution of samples according to gender and relationship between gender and affected side

Cleft side	Unilateral cleft lip		Unilateral cleft lip and alveolus		Unilateral cleft lip and palate	
Gender	Male	Female	Male	Female	Male	Female
Right	1	0	8	3	15	8
Left	–	–	10	8	49	19
Total	1	0	18	11	64	27
*P* value	–		0.355		0.535

**Table 4:** Distribution of samples according to cleft side

Cleft side	Unilateral cleft lip		Unilateral cleft lip and alveolus		Unilateral cleft lip and palate	
	Right	Left	Right	Left	Right	Left
Total	1	–	11	18	23	68
*P* value	–		0.264		0.001

**Table 5:** Number of missing teeth in unilateral cleft lip and alveolus and unilateral cleft lip and palate subjects

	Unilateral cleft lip and alveolus		Unilateral cleft lip and palate	
	Cleft side	Non-cleft side	Cleft side	Non-cleft side
Upper right central incisor	–	–	–	–
Upper left central incisor	–	–	–	–
Upper right lateral incisor	5	–	16	9
Upper left lateral incisor	9	–	39	2
Upper right 2nd premolar	1	–	1	4
Upper left 2nd premolar	–	–	3	2
Lower right central incisor	–	–	2	1
Lower left central incisor	–	–	1	2
Lower right lateral incisor	–	–	1	1
Lower left lateral incisor	–	–	1	1
Lower right 2nd premolar	–	–	–	2
Lower left 2nd premolar	–	–	–	3
Total	15	0	64	27

## Discussion

This study showed that unilateral and bilateral cleft lip and palate followed by unilateral cleft lip and alveolus were more common than other types of cleft in Persian population. Moreover, the incidence of cleft was significantly higher on the left side of unilateral cleft lip and palate patients. The findings of this study are similar to studies of other races. Fraser [14] reported the prevalence of left-sided clefts to be 66.6 %, and Wilson [15] reported it as 60 %. Kim and Baek [3] also found that patients had a significantly higher incidence on the left side than on the right. Their results showed that the prevalence of left-sided clefts in the unilateral cleft lip and palate patients was 67.4 %. While they did not find any significant difference in the distribution of cleft sidedness in unilateral cleft lip and alveolus patients.

Similar to the results of our study, hypodontia was found to occur more frequently on the cleft side than on the unaffected side [16]. Shapira et al. [17] also found that hypodontia of both the maxillary lateral incisors and second premolars were more frequent on the left side, which also had a higher frequency of clefting. In current study, substantially more missing teeth were detected in non-cleft side of unilateral cleft lip and alveolus patients. Similarly, Baek [5] and Kim also found considerably lower prevalence of hypodontia in the non-cleft side of these patients.

Ranta [18] reported that the upper lateral incisors are the most commonly missing teeth in the cleft area, followed by the second premolars in cleft lip and palate patients. These findings are similar to the findings of the current study for cleft lip and palate and cleft lip and alveolus patients. This finding has been explained by the proximity of the cleft to the lateral incisor region, which may strike and divide the primordial tissue related to the developing lateral incisor field [16].

One of the limitations of the current study which affects generalizing the results is the small number of patients. Further multi-center studies with a larger sample size and different races would definitely improve the literature. Future multidisciplinary studies focusing on genetic aspects of cleft patients in order to justify the higher prevalence of left-sided cleft are required.

## Conclusions

Current study showed that most cleft patients suffered from cleft lip and palate followed by unilateral cleft and alveolus. In this study, no differences were found in regard to the gender of the patients. The left side of the patients was affected substantially more than the right side. The frequency of the missing upper lateral incisors in the cleft side of the patients was significantly higher than the non-cleft side.
